# Prolonged Intestinal Obstruction

**Published:** 1883-07

**Authors:** 


### PROLONGED INTESTINAL OBSTRUCTION.

Dr. Heuster of Mobile, reports a remarkable and interesting case of intestinal obstruction of twenty-one days' standing, which was ultimately relieved by injections of carbonic acid gas. The patient was a woman, who had been confined after a tedious labor, accompanied by extensive perineal laceration, and followed by an attack of puerperal fever of three weeks' duration, and subsequently severe colic. The attack developed into a well-marked case of ileus. All the usual remedies, calomel and opium, warm poultices, injections of soap and water, or ox gall and water, morphia, extract of belladonna, etc., etc., failed to give relief. The stercoraceous vomiting steadily continued. On the seventeenth day it was determined to make an exploratory incision into the abdomen, but, owing to the difficulty in procuring assistance, the operation was postponed to the next day. Dr. Heuster then obtained a large siphon of seltzer water, attached an india-rubber tube, which he passed about eighteen inches up the bowel, and then turned on the gas. Before the bottle was half empty the fæces began to flow out, and when the flow stopped the gas was turned on again, to be interrupted by more fæces; and so it was kept up until the bottle was empty and the bowels too, apparently, from the quantity passed. After that the patient's stools became regular, and she had no further trouble with them. Dr. Heuster remarks that, as the exact seat of the obstruction could not be ascertained, its cause remained doubtful ; but he inclines to the opinion that the elastic and pervading force of carbonic acid gas thrown far up into the colon readily overcame the obstruction, and would appear to be the readiest means of treatment in similar cases.

